# Genetic Diversity of Symbiotic Green Algae of *Paramecium bursaria* Syngens Originating from Distant Geographical Locations

**DOI:** 10.3390/plants10030609

**Published:** 2021-03-23

**Authors:** Magdalena Greczek-Stachura, Patrycja Zagata Leśnicka, Sebastian Tarcz, Maria Rautian, Katarzyna Możdżeń

**Affiliations:** 1 Institute of Biology, Pedagogical University of Krakow, Podchorążych 2, 30-084 Kraków, Poland; magdalena.greczek-stachura@up.krakow.pl (M.G.-S.); patrycjazagata@gmail.com (P.Z.L.); 2 Institute of Systematics and Evolution of Animals, Polish Academy of Sciences, Sławkowska 17, 31-016 Krakow, Poland; tarcz@isez.pan.krakow.pl; 3 Laboratory of Protistology and Experimental Zoology, Faculty of Biology and Soil Science, St. Petersburg State University, Universitetskaya nab. 7/9, 199034 Saint Petersburg, Russia; mrautian@mail.ru

**Keywords:** *Paramecium bursaria* algal symbionts, chloroplast *3′rpl.36-5′infA* genes, nuclear ITS1-5.8S rDNA-ITS2, 28S rDNA sequence

## Abstract

*Paramecium bursaria* (Ehrenberg 1831) is a ciliate species living in a symbiotic relationship with green algae. The aim of the study was to identify green algal symbionts of *P. bursaria* originating from distant geographical locations and to answer the question of whether the occurrence of endosymbiont taxa was correlated with a specific ciliate syngen (sexually separated sibling group). In a comparative analysis, we investigated 43 *P. bursaria* symbiont strains based on molecular features. Three DNA fragments were sequenced: two from the nuclear genomes—a fragment of the ITS1-5.8S rDNA-ITS2 region and a fragment of the gene encoding large subunit ribosomal RNA (28S rDNA), as well as a fragment of the plastid genome comprising the 3′*rpl36*-5′*infA* genes. The analysis of two ribosomal sequences showed the presence of 29 haplotypes (haplotype diversity Hd = 0.98736 for ITS1-5.8S rDNA-ITS2 and Hd = 0.908 for 28S rDNA) in the former two regions, and 36 haplotypes in the 3′*rpl36*-5′*infA* gene fragment (Hd = 0.984). The following symbiotic strains were identified: *Chlorella vulgaris*, *Chlorella variabilis*, *Chlorella sorokiniana* and *Micractinium conductrix*. We rejected the hypotheses concerning (i) the correlation between *P. bursaria* syngen and symbiotic species, and (ii) the relationship between symbiotic species and geographic distribution.

## 1. Introduction

The unicellular ciliate *Paramecium bursaria* (Peniculia, Oligohymenophorea) is a host of endosymbiotic algal species. The mutualistic symbiosis exhibited by *P. bursaria* suppresses the genetic change of the inhabitant and ensures a nutritionally stable environment. Doebeli and Knowlton [1] reported that the rate of nucleotide substitutions was lower in symbiotic algae than in free-living relatives and their corresponding inhabitants since their co-evolution from an ancient association. *Paramecium* spp. usually comprise several sexually separated sibling groups, termed “syngens”, which are morphologically indistinguishable. Currently, *P. bursaria* strains have been assigned to five syngens (R1 to R5), which may correspond to some syngens described by Bomford [2,3]. Each syngen in Bomford’s collection (which was lost) had specific geographical distributions. Based on some similarities between syngens from the “old” and “new” collections, it has been suggested that syngen R1 is widespread in Europe; syngen R2 is widespread in Europe, extending eastwards to Siberia and Australia; syngen R4 is fairly widespread in the USA; and syngen R3 is present in Russia, Japan, China and the USA; finally, syngen R5 is represented by only four strains from two locations in western Europe [4].

Symbiotic algae isolated from different *Paramecium bursaria* syngens are represented by *Chlorella*-like species belonging to two genetically distinct “European” and “American” populations [5]. Gaponova et al. [6] confirmed the existence of two groups of symbionts based on the analysis of rDNA PCR products of two different lengths, which corresponded to the southern (three introns) or northern (single intron) group. Phylogenetic analyses based on the 28S rDNA gene, ITS 1, 5.8S rDNA and ITS 2 sequences suggested the existence of five different endosymbionts: *Chlorella vulgaris*, *Chlorella variabilis*, *Micractinium conductrix comb. nov.*, *Choricystis minor* (*Choriocystis parasitica comb. nov*.) and *Coccomyxa simplex*. Pröschold et al. [7] have confirmed the occurrence of two endosymbiont groups and found that *Micractinium conductrix* and *Chlorella vulgaris* belonged to the “European” population. Hoshina and Imamura [8] have found that *Chlorella vulgaris* is a symbiont of *Paramecium bursaria* strain. *Chlorella variabilis* represents the “American” population and has been found in *Paramecium bursaria* strains (CCAP211/84, 211/109 and 211/110) collected in the USA [7]. Algal symbionts of all *P. bursaria* strains of two different origins form one clade, but are split into two distinct lineages.

An evolutionary scenario for *P. bursaria* with respect to algal acquisition and subsequent switching assumes the coexistence of both species belonging to the “American” and “European” endosymbiont groups in one cell of ancestral *P. bursaria*. This sympatric relationship led to a continuous intron transmission. During evolution, the host “chose” one of the endosymbionts, and later “European” algae may have diverged into a lineage with a weakened host–algal partnership, in which accidental switching of the algae occurred twice [9,10].

Hoshina and Imamura [8] and Gaponova et al. [6] have shown that *P. bursaria* can contain different endosymbionts, depending on their origin. Nakahara et al. [11] identified an additional endosymbiont, *Choricystis minor*, in a strain from Florida (USA). Pröschold et al. [7] studied 17 strains of endosymbionts isolated from various hosts and different geographical locations. Phylogenetic analyses revealed that they were polyphyletic. The most studied ciliate, *P. bursaria,* harbors endosymbionts representing at least five different species: *Coccomyxa* sp., *Choricystis minor*, *Micractinium conductrix*, *Chlorella vulgaris* and *Chlorella variabilis*. *C. vulgaris*, *C. variabilis* and *Micractinium conductrix* are obligate endosymbionts of *P. bursaria* [7]. *M. tetrahymenae* forms a symbiotic association with *Tetrahymena utriculariae* only under anoxic or microaerobic conditions. Phylogenetic analyses using complex evolutionary models based on secondary structure have demonstrated that this endosymbiont represents a new species of *Micractinium*, which belongs to the so-called *Chlorella* clade (Trebouxiophyceae) [12].

In the present study, we investigated 43 strains of algal symbionts isolated from *P. bursaria* strains belonging to five syngens. The strains were collected in remote geographical locations. Twenty sequences of symbionts were available in GenBank (28S rDNA and ITS1-5.8S rDNA-ITS2 fragment). The strains of *Coccomyxa chodatii*, *Stigeoclonium tenue*, *Stigeoclonium variabile*, *Parachlorella kessleri* and *Actinastrum hantzschii* were used as outgroups. Three loci: a fragment of the ITS1-5.8S rDNA-ITS2 region and a fragment 28S rDNA, as well as chloroplast genes encoding ribosomal protein L36 (*rpl36*) and translation initiation factor IF-1 (*infA*) were applied to study phylogenetic relationships of symbiotic algae. The selected ribosomal primers were specific to symbiotic cells, which did not allow the simultaneous amplification of *P. bursaria* rDNA fragments. The 28S rDNA is characterized by higher variability than the 18S rDNA [8]. The ITS1-5.8S rDNA-ITS2 region is highly variable among the sequences of different species, while it is relatively conserved among the sequences of the same species of algae. Furthermore, this fragment is most commonly available in GenBank, which facilitates comparative analysis. The 3′*rpl36*-5′*infA* gene fragment has been selected due to the presence of an intergenic region, which is suspected to have more potential substitution sites than the gene-coding regions.

The main aim of the study was to determine the molecular phylogenetic relationships among green algal endosymbionts of *P. bursaria* in order to explore the history of the symbiosis events. We tried to answer whether endosymbiosis of a green algae in the host *P. bursaria* took place prior to the diversification of the host lineage into the various syngens or if endosymbionts are incorporated over and over again. In the latter case we assess whether endosymbionts are host-specific or if there is no relationship between host syngens and endosymbiont lineage.

## 2. Results

### 2.1. Syngen Identification

Identification of *Paramecium bursaria* syngens was performed by mating the studied strain with standard strains representing all mating types of each syngen. The number of symbiotic strains of algal species identified in each of the five *P. bursaria* syngens is presented in Table 1.

### 2.2. Geographical Distribution of Paramecium Bursaria Symbionts

**Figure 1:**
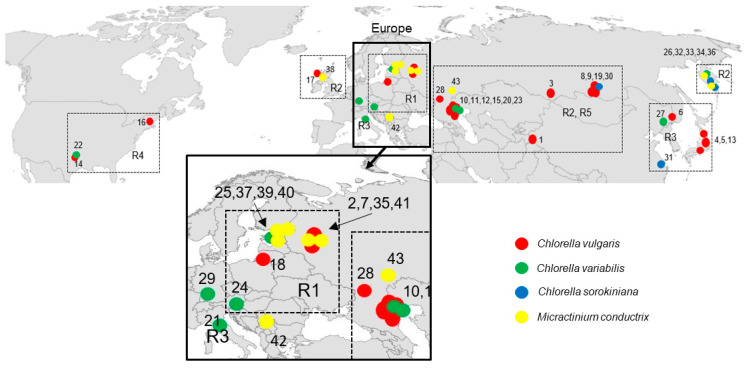
Geographical distribution of *Paramecium bursaria* symbionts with numbers corresponding to those in Table 2.

*P. bursaria* syngens and their geographical distribution are shown in Figure 1 and Table 2. Syngen R1 from central Asia (Tajikistan) harbored *C. vulgaris* strain but those from Europe (Wien) contained *C. variabilis.* Endosymbiotic *Micractinium conductrix* was isolated from the syngen originating from north-eastern Europe (St. Petersburg, Tver). Syngen R2 of *P. bursaria* was collected most frequently, and 10 endosymbionts from central Asia (Altai, Lake Baikal), eastern Europe (Astrakhan), eastern Europe (Tver, Yaroslavl, Kaliningrad), and Scotland (Europe) were assigned to *C. vulgaris*. Four strains from eastern Europe (Astrakhan), Far East (Kamchatka) and from Germany (Europe) belonged to *C. variabilis*. Two strains from Kamchatka and one from central Asia (Lake Baikal) were assigned to *C. sorokiniana.* Seven strains of *Micractinium conductrix* from Asia and Europe were found in this syngen. Green endosymbionts from syngen R3 sampled in Japan and Far East (Khabarovsk) belonged to the *C. vulgaris* clade, but *C. variabilis* (Khanka Nature Reserve) and *C. sorokiniana* strains were also found in China. One strain of *C. variabilis* was isolated in Europe (Italy). Strains isolated from syngen R4 of *P. bursaria* originating from the USA were assigned to *C. vulgaris* and *C. variabilis*. Endosymbionts isolated from syngen R5 originating from eastern Europe (Astrakhan) were assigned to *C. vulgaris*, while the strain isolated from the same *P. bursaria* syngen sampled in north-eastern Europe (St. Petersburg) was *C. variabilis*.

### 2.3. Molecular Results

Results of the analysis of ITS1-5.8S-rDNA-ITS2, 28S rDNA and 3′*rpl36*-5′*infA* chloroplast gene fragments revealed similarity of the isolated strains to the species described as *Chlorella vulgaris*, *Chlorella variabilis*, *Chlorella sorokiniana* and *Micractinium conductrix*. Phylogenetic inference showed that these strains belonged to four distinct clades, thus the endosymbionts were polyphyletic.

#### 2.3.1. Analysis of the ITS1-5.8S rDNA-ITS2 Fragment

Results of the analysis of the ITS1-5.8S rDNA-ITS2 fragments (543 bp) of 37 endosymbionts revealed the existence of 29 haplotypes in the studied dataset. The value of the interspecific haplotype diversity was Hd = 0.987 and the nucleotide diversity was π = 0.16040. Nucleotide frequencies were as follows: A = 20.5%, T = 22.6%, C = 30.1% and G = 26.8%.

**Figure 2:**
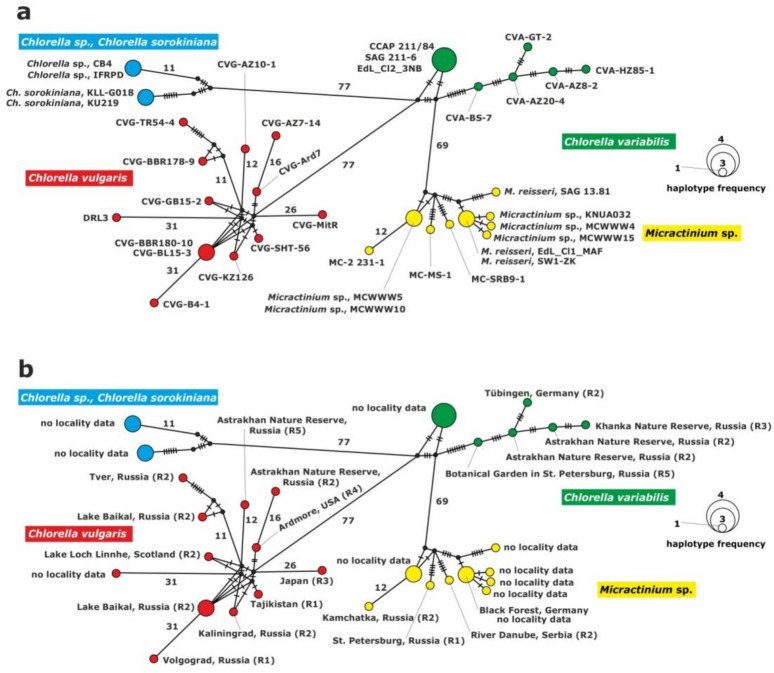
Haplotype network constructed for 37 symbiotic alga strains based on the comparison of the ITS1-5.8S rDNA-ITS2 sequences, (**a**) with strain abbreviations, (**b**) geographical origin of *P. bursaria* strains and syngens. The size of the dots is proportional to haplotype frequency. Median vectors that represent hypothetical intermediates or unsampled haplotypes are shown as black dots. Hatch marks on individual branches represent nucleotide substitutions between individual haplotypes (corresponding number was assigned for more than 10). Haplotypes marked as “no locality data” were acquired from GenBank.

The haplotype network of the ITS1-5.8S rDNA-ITS2 fragment was constructed for the inference and visualization of genetic relationships between green endosymbionts of *P. bursaria* (Figure 2). Four haplogroups were identified for the rDNA fragment in the studied strains, i.e., *C. vulgaris*, *C. variabilis*, *C. sorokiniana* and *M. conductrix*. The clade of *C. vulgaris* was composed of 12 haplotypes; one of them comprised two strains isolated from *P. bursaria* syngen R2: CVG-BBR-180-10 and CVG-BL15-3 sampled from the Baikal Lake (central Asia). The clade of *C. variabilis* included six haplotypes. Three strains: CCAP 211/84, SAG 211-6 and Edl_Cl2_3NB from GenBank formed a common haplotype. The remaining strains represented single haplotypes.

The clade of *C. sorokiniana* was composed of two unique haplotypes. The first one consisted of two *Chlorella* sp. strains, CB4 and IFRPD, and the second one of *Chlorella sorokiniana* KLL-G018 and KU219 from GenBank.

The following clade, *Micractinium,* included nine haplotypes and seven of them represented unique haplotypes; two of them were composed of two strains: *Micractinium* sp., MCWWW5 and MCWWW10 from GenBank, and the second haplotype: *Micractinium reisseri* EDL_Cl1_MAF from GenBank and SW1-ZK1 from Germany. There were 88 to 112 differences between *C. variabilis* and *C. sorokiniana*, 81 to 128 between *C. vulgaris* and *C. variabilis*, 72 to 100 between *C. variabilis* and *Micractinium*, 149 to 192 between *Micractinium* and *C. vulgaris* and 168 to 204 differences between *C. vulgaris* and *C. sorokiniana*. Intraspecific variation among haplotypes was the result of several substitutions (Table 2, Figure 2).

#### 2.3.2. Analysis of the 28S rDNA Fragment

Results of the analysis of 28S rDNA fragments (555 bp) of 43 symbionts isolated from different *P. bursaria* strains showed the presence of 29 haplotypes. The value of the interspecific haplotype diversity was Hd = 0.908 and the nucleotide diversity was π = 0.03165. Nucleotide frequencies were as follows: A = 26.7%, T = 18.7%, C = 23.8% and G = 30.8%.

**Figure 3:**
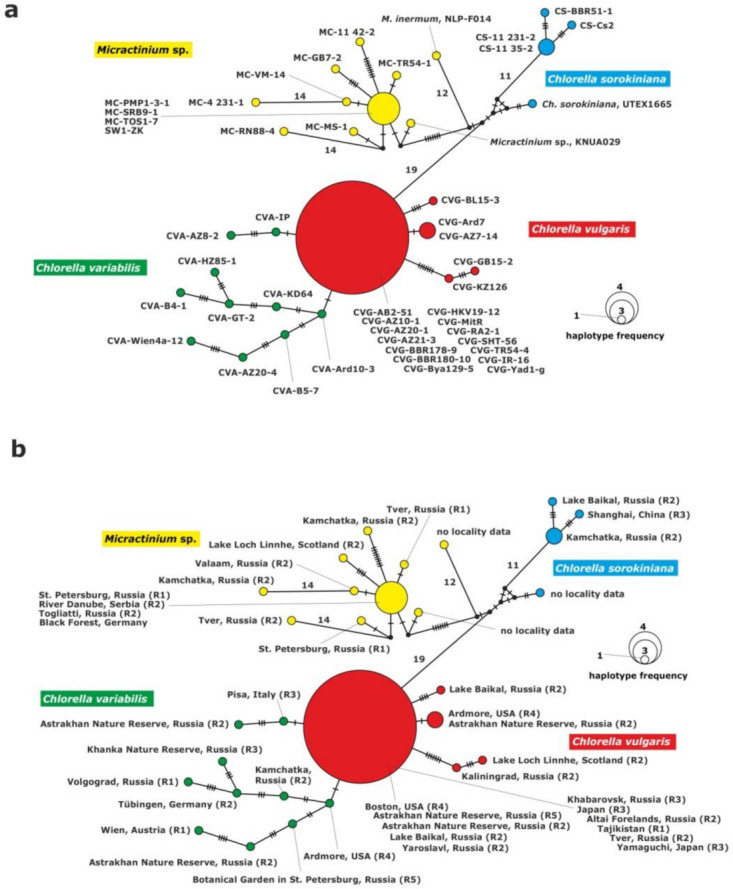
Haplotype network constructed for 43 symbiotic algae of *P. bursaria* strains based on sequence comparison of the 28S rDNA gene fragment, (**a**) with strain abbreviations, (**b**) geographical origin of *P. bursaria* strains and syngens. The size of the dots is proportional to haplotype frequency. Median vectors that represent hypothetical intermediates or un-sampled haplotypes are shown as black dots. Hatch marks on individual branches represent nucleotide substitutions between individual haplotypes (corresponding number was assigned for more than 10). Haplotypes marked as “no locality data” were acquired from GenBank.

The haplotype network of the 28S rDNA fragment grouped the strains into four clades: *C. vulgaris*, *C. variabilis*, *C. sorokiniana* and *Micractinium*. The clade of *C. variabilis* was composed of 10 unique haplotypes with 2 to 9 substitutions between them (Figure 3).

The *C. vulgaris* clade consisted of five unique haplotypes. One of them included 14 strains: CVG-Bya129-5 (Yaroslavl) and CVG-TR54-4 (Tver) from eastern Europe, CVG-SHT56 (Tajikistan) from central Asia, CVG-RA2-1 (Altai) and CVG-BBR178-9, CVG-BBR180-10 (Baikal Lake) from central Asia, CVG-AZ10-1, CVG-AZ20-1, CVG-AZ21-3, (Astrakhan) from eastern Europe, CVG-HKV19-12 (Khabarovsk) from the Far East, CVG-JR-16, CVG-MitR and CVG-Yad1-g from Japan, CVG-AB2-51 (Boston) from USA. The second haplotype was composed of two strains: CVG-AZ7-14 (Astakhan) from eastern Europe and CVG-Ard7 (Ardmore) from USAj. The other haplotypes were represented by the following single strains: CVG-BL15-3, CVG-KZ-126 and CVG-GB15-2. The *C. variabilis* clade was composed of 10 single strains.

The *Micractinium* clade was composed of 10 haplotypes. One of them included four strains from Europe: MC-PMP1-3-1, (St. Petersburg, north-eastern Europe), MC-SRB9-1 (Serbia, southern Europe), MC-TOS1-7 (Togliatii, south-eastern Europe) and SW1-ZK (Germany, western Europe). The other nine corresponded to single strains: MC-4 231-1, MC-VM-14, MC-RN88-4, MC-MS-1, MC-GB7-2, MC-11 42-2, MC-TR54-1, NLP-F014 and KNUA029.

The last clade consisted of *C. sorokiniana* representatives, and included four haplotypes. One haplotype was formed by two strains from the Far East origin: CS-11 231-2 and CS-11 35-2 (Kamchatka) and the other two represented single strains: CS-BBR51-1 and CS-Cs2.

Interspecific variability was higher when *C. vulgaris* to *Micractinium* or *C. variabilis* to *Micractinium* were compared (28–58 differences). There was a low number of substitutions between *C. vulgaris* and *C. variabilis* (1–20 differences) (Table 2, Figure 3).

#### 2.3.3. Analysis of the rpl36-infA Genes Fragment

Results of the *rpl36-infA* gene fragment (267 bp) analysis in symbionts isolated from 43 *P. bursaria* strains showed the presence of 36 haplotypes. The value of the interspecific haplotype diversity was Hd = 0.984, and the nucleotide diversity was π = 0.07886. Nucleotide frequencies were as follows: A = 29.6%, T = 36.0%, C = 18.5% and G = 15.9%.

**Figure 4:**
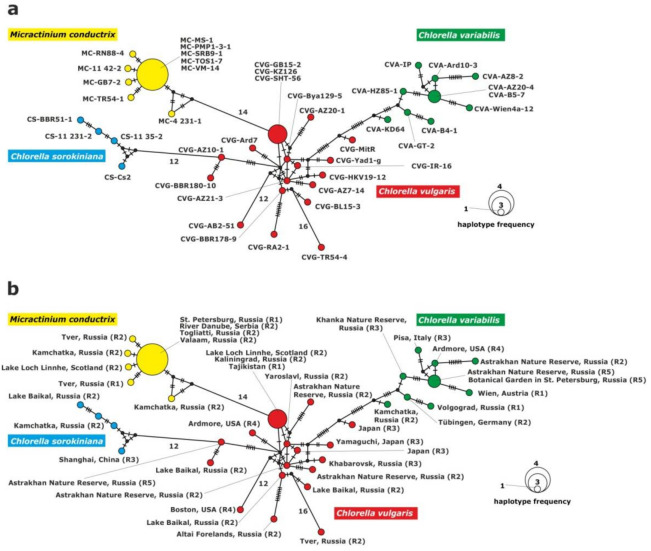
Haplotype network constructed for 43 symbiotic algae strains based on the comparison of the 3′*rpl36*-5′*infA* genes sequences, (**a**) with strain abbreviations, (**b**) geographical origin of *P. bursaria* strains and syngens. The size of the dots is proportional to haplotype frequency. Median vectors that represent hypothetical intermediates or unsampled haplotypes are shown as black dots. Hatch marks on individual branches represent nucleotide substitutions between individual haplotypes (corresponding number was assigned for more than 10).

The haplotype network of chloroplast gene fragments grouped the strains into four clades: *C. vulgaris*, *C. variabilis*, *C. sorokiniana* and *M. conductrix* (Figure 4). The *C. vulgaris* clade included 17 haplotypes; one haplotype was represented by three strains. Two strains from Europe: CVG-GB15-2 (Scotland), CVG-KZ-126 (Kaliningrad) isolated from *P. bursaria* syngen R2, and one strain from central Asia: CVG-SHT-56 (Tajikistan) from syngen R1. The remaining haplotypes consisted of single strains.

The clade of *C. variabilis* consisted of nine haplotypes and eight of them included single strains. Strain CVA-B5-7 (St. Petersburg, north-eastern Europe) from syngen R5 and strain CVA-AZ20-4 (Astrakhan, eastern Europe) from syngen R2 belonged to the ninth haplotype.

The *C. sorokiniana* clade was composed of four unique haplotypes corresponding to single strains.

The *M. conductrix* clade included six haplotypes, five of them represented single strains and one haplotype was composed of the five following strains: MC-PMP1-3-1 and MC-MS-1 (St. Petersburg, north-eastern Europe), isolated from syngen R1, MC-SRB9-1 (Serbia, southern Europe), MC-TOS1-7 (Togliatii, south-eastern Europe), and MC-VM-14 (Valaam, northern Europe) isolated from syngen R2.

There were 18 to 43 substitutions between *C. vulgaris* and *C. variabilis*, 19 to 49 substitutions between *C. vulgaris* and *C. sorokiniana*, 41 to 51 between *C. sorokiniana* and *M. conductrix,* and 35 to 57 substitutions between *M. conductrix* and *C. variabilis* (Table 2, Figure 4).

## 3. Discussion

*Paramecium bursaria* is an archetypical outbreeder, which presumably means that its effective population size is large. *P. bursaria* is divided into five syngens which are characterized by a specific geographical distribution. Nyberg [22] concluded that *P. bursaria* syngens, as extreme outbreeders, should be globally distributed, but Bomford [2] and Greczek-Stachura et al. [4] postulated that most sibling species were restricted to certain geographical regions, and thus adapted to specific conditions. Based on the comparison of syngens from Bomford’s collection and new syngen annotations, it is known that syngens R3 and R4 have been found in the United States [23], and syngen R3 has been reported later in China [24]. According to the study by Hoshina et al. [25], *P. bursaria* strains from Japan were also classified as syngen R3. Two syngens, R1 and R2, are only of Eurasian origin, and have been recorded at various locations from Great Britain to central Siberia; in addition, two strains of syngen 2 have been found in one locality in Australia. Syngen R3 strains have been isolated in far-eastern Russia and south-eastern Siberia (but never western Siberia), China, Japan, and the USA. Recently, this syngen has been reported in Europe, namely in Austria and in Italy (although the strain from Pisa was collected in a botanical garden, where it could have been brought along with some tropical plants). Syngen 4 strains are restricted to the USA. Strains belonging to syngen 5 have been found in the Volga delta, known for its great migration routes of waterfowl that are suspected transmitters of paramecia [4,26]. The current investigation of different syngens of *P. bursaria* collected in Europe, Asia and North America confirmed the previous knowledge about their biogeography. *P. bursaria* syngen R1 has been found in central Asia and north-eastern Europe. Strains of syngen R2 have been found in Asia and Europe. Syngen R3 was sampled in Japan, Far East and China. Strains of syngen R4 originate from the USA and syngen R5 strains are derived from eastern Europe and north-eastern Europe (Figure 1, Table 2).

The existence of syngens is the result of the process of speciation. The key question regarding evolution is: what are the driving forces behind initial speciation of *Paramecium bursaria?* Geographic isolation is often the main speciation factor, but its significance in protists is uncertain as there is still disagreement over their distribution—whether it is cosmopolitan or endemic.

If *P. bursaria* syngens are hosting the same species of endosymbiotic algae, they can be sympatric or other speciation mechanisms may play a leading role. Therefore, in our opinion, identification of species of endosymbiotic algae can explain a possible process of co-evolution. In the present study, we have identified four species of endosymbiotic algae, i.e., *C. vulgaris*, *C. variabilis*, *C. sorokiniana* and *M. conductrix*. Spanner et al. [27], based on ITS-2 sequencing, identified *Chlorella variabilis* and *Micractinium conductrix* in *Paramecium bursaria* cells. The two above endosymbionts have been identified in strains belonging to syngens R1 and R2 of *P. bursaria*, which originated from Europe. Moreover, we have found *C. vulgaris* and *C. variabilis* in all five syngens of *P. bursaria*, *M. conductrix* was present in syngen R1 and R2, and *C. sorokiniana* in syngen R2 and R3 (Table 1). Gaponova et al. [6] have also found *M. conductrix* in *P. bursaria* isolates collected in North Karelia (Russia). Overall, it seems that *M. conductrix* occurs only in Europe, whereas *C. variabilis* is distributed worldwide. Hoshina et al. [5,10] established the geographical distribution of *Micractinium* sp. in the regions of England, Germany, Austria and northern Karelia, which was consistent with the results obtained by Luo et al. [17,28]. Strains belonging to the American group derived from USA, Japan, China and southern Australia carried symbiotic algae classified as *Chlorella vulgaris* and *Chlorella variabilis* [7]. Hoshina and Imamura [9] identified the strains from Kaliningrad as *C. vulgaris*, similar to our findings i.e., the strain isolated from syngen R2. Pröschold et al. [7] have suggested that *C. variabilis* is characteristic of the American but not the European group; however, according to our results, the strains from St. Petersburg and Valaam as well as strains from central Europe (Pisa, River Danube in Serbia) have been assigned to *C. variabilis* and *M. conductrix*.

Our findings suggests that there is no correlation between *P. bursaria* syngen and the species of symbiont, as was previously argued by Weis [29]. Similarly, Reisser et al. [30] stated that *P. bursaria* strains of American or European origin formed a stable symbiosis with symbionts of both groups. Then, Meier and Wiessner [31] demonstrated that *P. bursaria* could eliminate symbionts and subsequently be reinfected by new symbionts. Summerer et al. [32] mixed two aposymbiotic *P. bursaria* strains with symbiotic and free-living *Chlorella* strains. Symbioses were formed with endosymbiotic *Chlorella*, with the exception of those from *H. viridis* and free-living algae. Similarly, in the current survey we demonstrated that there is no strong relationship between species of symbionts and the geographical distribution of their host, *P. bursaria.* This may be explained by the ancestral aposymbiotic ciliate *P. bursaria* possibly having acquired different species of green algae and later diverging into a lineage with a host-algal partnership where accidental algal change may have occurred. Summerer et al. [33] analyzed nuclear 18S rDNA, the ITS1 region and chloroplast 16S rDNA from algal symbionts of *P. bursaria* strains originating from two lakes in Austria. These strains formed a clade with two distinct lineages, suggesting the existence of a biogeographic pattern. Genetic differences between symbiotic algae are 10 times higher than between free-living algae. This suggests that multiple symbiotic origins are more likely than the divergence of one symbiotic species to different symbiotic algae existing currently [25]. The endosymbiotic lifestyle has evolved many times in green algae, as evidenced by the presence of numerous haplotypes of endosymbiotic algae in the haplotype network based on the nuclear ITS1-5.8S rDNA-ITS2 fragment, 28S rDNA fragment and 3′*rpl36*-5′*infA* gene sequences. Endosymbionts of the Chlorellaceae species, which also serve as specific hosts for large dsDNA viruses known as chloroviruses, do not cluster together, providing strong evidence for independent transitions to endosymbiosis [34].

Therefore, we suppose that the speciation of *P. bursaria* syngens was an earlier evolutionarily event than the establishment of symbiosis, as evidenced by the diversity of symbionts and their lack of specificity.

## 4. Materials and Methods

### 4.1. Strain Cultivation and Strain Crosses

*Paramecium bursaria* strains were cultivated on a lettuce medium according to Sonneborn [35], fed *Klebsiella pneumoniae* (SMC) and stored at 18 °C (12L/12D). We investigated 43 symbiotic strains isolated from *P. bursaria* cells derived from different geographical locations. We also analyzed 20 sequences of symbiotic algae available in GenBank and strains of *Coccomyxa chodatii*, *Stigeoclonium tenue*, *Stigeoclonium variabile*, *Parachlorella kessleri* and *Actinastrum hantzschii* as outgroups (Table 2).

Identification of *P. bursaria* syngens was performed by mating reaction of a studied strain with standard strains representing all mating types of each syngen. The studied strains were assigned to a certain syngen based on the occurrence of strong clumping at the beginning of the mating reaction, the presence of mating couples and survival of F_1_ progeny.

### 4.2. Molecular Methods

Symbiotic DNA was extracted using the GeneJET Plant Genomic DNA Purification Kit (ThermoScientific) according to the protocol. Dense *P. bursaria* culture (1.5 mL) was harvested from a liquid culture by centrifugation. Then, the pellet was sonicated on ice for 10 s at 40 W. Subsequently, the standard extraction protocol was followed. The ITS1-5.8S rDNA-ITS2 fragment was amplified using the following primers pairs: ITS1 [32]/ITS2R (primer designed for the present study, Table 3) and ITS1F/ITS2R (primers designed for the present study, Table 3) according to the protocol with the following parameters: initial denaturation at 95 °C for 5 min followed by 30 cycles of denaturation at 95 °C for 1 min, annealing at 54 °C for 2 min, extension at 72 °C for 3 min and a final extension at 72 °C for 5 min.

The fragment of a 28S rDNA was amplified by polymerase chain reaction (PCR) using the HLR0F/HLR4R primer pair [8,37] (Table 3), according to the protocol described by Hoshina et al. [38]. The fragment of 3′*rpl36*-5′*infA* genes was amplified using the UCP2F and UCP2R primer set (Table 3), according to Provan et al. [36]. After amplification, PCR products were separated by electrophoresis in 1% agarose gel for 1 h at 95 V and then gel-purified using NucleoSpin Extract II (Macherey-Nagel, Düren, Germany). Sequencing reaction was performed in both directions using the BigDye Terminator v3.1 kit (Applied Biosystems, Foster City, USA). Sequencing products were precipitated using Ex Terminator (A&A Biotechnology, Gdynia, Poland).

### 4.3. Data Analyzes

Sequences were examined and corrected using Chromas Lite (Technylesium), and aligned using BioEdit [39]. The analysis of haplotype diversity (Hd) and nucleotide diversity (π) was carried out using DnaSP v5.10.01 [39]. The analysis of nucleotide frequencies and identification of the best nucleotide substitution models for maximum likelihood tree reconstruction (T92 + G for three loci) were conducted using Mega v5.1. Haplotype networks were constructed using the Median Joining method implemented in the Network 4.6.1.3 software [40,41].

## 5. Conclusions

The ITS1-5.8S rDNA-ITS2 fragment is the most appropriate molecular marker to identify and resolve evolutionary relationship between symbionts of *Paramecium bursaria*. We assigned symbiotic algae of *P. bursaria* to four species: *Chlorella vulgaris*, *Chlorella variabilis*, *Chlorella sorokiniana* and *Micractinium conductrix*. The division of *P. bursaria* endosymbionts into the American and European groups and the correlation between *P. bursaria* syngen and a symbiotic species has not been confirmed. No strong relationships have been found between symbiotic species and geographical distribution of their host *P. bursaria*.

Molecular markers: ITS1-5.8S rDNA-ITS2, 28S rDNA fragments and 3′*rpl36*-5′*infA* gene fragments are useful molecular tools for distinguishing closely related taxa of *P. bursaria* symbionts. The ITS1-5.8S rDNA-ITS2 fragment is the most appropriate due to its high interspecific and low intraspecific variability. Additionally, the application of two independent genome fragments (nuclear and chloroplast) increases the reliability of the results.

## Figures and Tables

**Table 1:** The number of symbiotic strains of particular algal species identified in five syngens of *Paramecium bursaria.*

Endosymbiont Species	Syngen of *Paramecium bursaria*				
	R1	R2	R3	R4	R5
*Chlorella vulgaris*	2	10	4	1	1
*Chlorella variabilis*	1	4	2	1	1
*Chlorella sorokiniana*	0	3	1	0	0
*Micractinium conductrix*	3	7	0	0	0

**Table 2:** Strains of symbiotic algae studied in the current survey.

No.	Algal (Endosymbiont) Species	Algal (Endosymbiont) Strain	*Paramecium bursaria* (Host) Strain	Taxonomic Designation of the Host	Origin of the Host	GenBank Accession Number			References
						28S rDNA	3′*rpl36*-5′*infA*	ITS1-5.8S-ITS2	
1.	*Chlorella vulgaris*	CVG-SHT-56	SHT-56	R1	Tajikistan	KX639563	KX639603	KX639535	This study
2.	*Chlorella vulgaris*	CVG-TR54-4	TR54-4	R2	Tver, Russia	KX639564	KX639604	KX639536	This study
3.	*Chlorella vulgaris*	CVG-RA2-1	RA2-1	R2	Altai Forelands, Russia	KX639562	KX639602	nd	This study
4.	*Chlorella vulgaris*	CVG-MitR	MitR	R3	Japan	KX639561	KX639601	KX639534	This study
5.	*Chlorella vulgaris*	CVG-JR-16	JR-16	R3	Japan	KX639560	KX639600	nd	This study
6.	*Chlorella vulgaris*	CVG-HKV19-12	HKV19-12	R3	Khabarovsk, Russia	KM203671	KM203663	nd	[13]
7.	*Chlorella vulgaris*	CVG-Bya129-5	Bya129-5	R2	Yaroslavl, Russia	KX639559	KX639598	nd	This study
8.	*Chlorella vulgaris*	CVG-BBR180-10	BBR180-10	R2	Lake Baikal, Russia	KX639557	KX639596	KX639531	This study
9.	*Chlorella vulgaris*	CVG-BBR178-9	BBR178-9	R2	Lake Baikal, Russia	KX639556	KX639595	KX639530	This study
10.	*Chlorella vulgaris*	CVG-AZ21-3	AZ21-3	R2	Astrakhan Nature Reserve, Russia	KX639555	KX639594	nd	This study
11.	*Chlorella vulgaris*	CVG-AZ20-1	AZ20-1	R5	Astrakhan Nature Reserve, Russia	KX639554	KX639593	nd	This study
12.	*Chlorella vulgaris*	CVG-AZ10-1	AZ10-1	R5	Astrakhan Nature Reserve, Russia	KM203670	KM203662	KX639528	[13], this study (ITS1-5.8S-ITS2)
13.	*Chlorella vulgaris*	CVG-Yad1-g	Yad1-g	R3	Yamaguchi, Japan	KX639565	KX639605	nd	This study
14.	*Chlorella vulgaris*	CVG-Ard7	Ard7	R4	Ardmore, USA	KX639552	KX639591	KX639526	This study
15.	*Chlorella vulgaris*	CVG-AZ7-14	AZ7-14	R2	Astrakhan Nature Reserve, Russia	KX639553	KX639592	KX639527	This study
16.	*Chlorella vulgaris*	CVG-AB2-51	AB2-51	R4	Boston, USA	KM203673	KM203661	nd	[13]
17.	*Chlorella vulgaris*	CVG-GB15-2	GB15-2	R2	Lake Loch Linnhe, Scotland	KX639551	KX639599	KX639525	This study
18.	*Chlorella vulgaris*	CVG-KZ-126	KZ-126	R2	Kaliningrad, Russia	KM203672	KM203660	KX639533	[13], this study (ITS1-5.8S-ITS2)
19.	*Chlorella vulgaris*	CVG-BL15-3	BL15-3	R2	Lake Baikal, Russia	KX639558	KX639597	KX639532	This study
20	*Chlorella vulgaris*	CVG-B4-1	B4-1	R1	Volgograd, Russia	KX639546	KX639586	KX639529	This study
21.	*Chlorella variabilis*	CVA-AZ8-2	AZ8-2	R2	Astrakhan Nature Reserve, Russia	KX639544	KX639584	KX639520	This study
22.	*Chlorella variabilis*	CVA-IP	IP	R3	Pisa, Italy	KX639549	KX639589	nd	This study
23.	*Chlorella variabilis*	CVA-Ard10-3	Ard10-3	R4	Ardmore, USA	KM203667	KM203658	nd	[13]
24.	*Chlorella variabilis*	CVA-AZ20-4	AZ20-4	R2	Astrakhan Nature Reserve, Russia	KX639545	KX639585	KX639521	This study
25.	*Chlorella variabilis*	CVA-Wien4a-12	Wien4a-12	R1	Wien, Austria	KX639550	KX639590	nd	This study
26.	*Chlorella variabilis*	CVA-B5-7	B5-7	R5	Botanical Garden in St. Petersburg, Russia	KM203669	KM203659	KX639522	[13], this study (ITS1-5.8S-ITS2)
27.	*Chlorella variabilis*	CVA-KD64	KD64	R2	Kamchatka, Russia	KM203668	KM203657	nd	[13]
28.	*Chlorella variabilis*	CVA-HZ85-1	HZ85-1	R3	Khanka Nature Reserve, Russia	KX639548	KX639587	KX639524	This study
29.									
30.	*Chlorella variabilis*	CVA-GT-2	GT-2	R2	Tübingen, Germany	KX639547	KX639587	KX639523	This study
31.	*Chlorella sorokiniana*	CS-BBR51-1	BBR51-1	R2	Lake Baikal, Russia	KX639542	KX639582	nd	This study
32.	*Chlorella sorokiniana*	CS-Cs2	Cs2	R3	Shanghai, China	KX639543	KX639583	nd	This study
33.	*Chlorella sorokiniana*	CS-11 231-2	11 231-2	R2	Kamchatka, Russia	KX639540	KX639580	nd	This study
34.	*Chlorella sorokiniana*	CS-11 35-2	11 35-2	R2	Kamchatka, Russia	KX639541	KX639581	nd	This study
35.	*Micractinium conductrix*	MC-11 42-2	11 42-2	R2	Kamchatka, Russia	KX639567	KX639574	nd	This study
36.	*Micractinium conductrix*	MC-RN88-4	RN88-4	R2	Tver, Russia	KX639570	KX639577	nd	This study
37.	*Micractinium conductrix*	MC-4 231-1	4 231-1	R2	Kamchatka, Russia	KX639566	KX639573	KX639537	This study
38.	*Micractinium conductrix*	MC-MS-1	MS-1	R1	St. Petersburg, Russia	KM203675	KM203666	KX639538	[13], this study (ITS1-5.8S-ITS2)
39.	*Micractinium conductrix*	MC-GB7-2	GB7-2	R2	Lake Loch Linnhe, Scotland	KX639568	KX639575	nd	This study
40.	*Micractinium conductrix*	MC-VM-14	VM-14	R2	Valaam, Russia	KM203674	KM203664	nd	[13]
41.	*Micractinium conductrix*	MC-PMP1-3-1	PMP1-3-1	R1	St. Petersburg, Russia	KX639569	KX639576	nd	This study
42.	*Micractinium conductrix*	MC-TR54-1	TR54-1	R1	Tver, Russia	KX639572	KX639579	nd	This study
43.	*Micractinium conductrix*	MC-SRB9-1	SRB9-1	R2	River Danube, Serbia	KX639571	KX639578	KX639539	This study
44.	*Micractinium conductrix*	MC-TOS1-7	TOS1-7	R2	Togliatti, Russia	KM203676	KM203665	nd	[13]
45.	*Micractinium inermum*	NLP-F014	nd	nd	nd	KF597304.1	nd	nd	Unpublished data
46.	*Chlorella sorokiniana*	UTEX 1665	nd	nd	nd	KJ676113.1	nd	nd	[14]
47.	*Micractinium* sp.	KNUA029	nd	nd	nd	KM243321.1	nd	nd	[15]
48.	*Micractinium reisseri* (*conductrix*)	SW1-ZK, (SW1)	nd	nd	Black Forest, Germany	AB437256.1	nd	AB437244.1	[10]
49.	*Micractinium* sp.	MCWWW15	nd	nd	nd	nd	nd	KP204593.1	[16]
50.	*Micractinium* sp.	MCWWW4	nd	nd	nd	nd	nd	KP204582.1	[16]
51.	*Micractinium* sp.	MCWWW5	nd	nd	nd	nd	nd	KP204583.1	[16]
52.	*Micractinium* sp.	MCWWW10	nd	nd	nd	nd	nd	KP204588.1	[16]
53.	*Micractinium* sp.	MCWWW11	nd	nd	nd	nd	nd	KP204589.1	[16]
54.	*Micractinium* sp.	KNUA032	nd	nd	nd	nd	nd	KM243324.1	[15]
55.	*Micractinium reisseri* (*conductrix*)	EdL_Cl1_MAF	nd	nd	nd	nd	nd	KF887345.1	Unpublished data
56.		SAG 13.81	nd	nd	nd	nd	nd	FM205866.1	[17]
57.	*Chlorella* sp.	CB4	nd	nd	nd	nd	nd	JQ710683.1	Unpublished data
58.	*Chlorella* sp.	IFRPD	nd	nd	nd	nd	nd	AB260898.1	[8]
59.	*Chlorella sorokiniana*	KLL-G018	nd	nd	nd	nd	nd	KP726221.1	[18]
60.	*Chlorella sorokiniana*	KU219	nd	nd	nd	nd	nd	KM061463.1	Unpublished data
61.	*Chlorella variabilis*	CCAP 211/84	nd	nd	nd	nd	nd	FN298923.1	[7]
62.	*Chlorella variabilis*	SAG 211-6	nd	nd	nd	nd	nd	FM205849.1	[17]
63.	*Chlorella variabilis*	EdL_Cl2_3NB	nd	nd	nd	nd	nd	KF887350.1	Unpublished data
64.	*Chlorella vulgaris*	DRL3	nd	nd	nd	nd	nd	JX139000.1	Unpublished data
65.	*Coccomyxa chodatii*	SAG: 216-2	nd	nd	nd	HG972989.1	nd	nd	[19]
66.	*Stigeoclonium tenue*	CCAP 477/11A	nd	nd	nd	HF920680.1	nd	nd	[20]
67.	*Stigeoclonium variabile*	CCAP 477/13	nd	nd	nd	HF920679.1	nd	nd	[20]
68.	*Parachlorella kessleri*	SAG: 211-11g	nd	nd	nd	nd	X65099.1	nd	[21]
69.	*Actinastrum hantzschii*	SAG 2015	nd	nd	nd	nd	nd	FM205841.1	[18]

**Table 3:** Primers used in the present study.

DNA Fragment	Primer	Sequence 5′-3′	References
ITS1-5.8S rDNA-ITS2	ITS1	TCCGTAGGTGAACCTGCGG	[33]
	ITS1F	AATCTATCGAATCCACTTTGGTAAC	Designed in the present study
	ITS2R	CTGCTAGGTCTCCAGCAAAG	Designed in the present study
28S rDNA frgment	HLR0F	GGCAAGACTACCCGCTGAA	[8]
	HLR4R	TTTCAAGACGGGCCGATT	[8]
3′*rpl36*-5′*infA* genes	UCP2F	CCTTGWCKTTGTTTATGTTTKGG	[36]
	UCP2R	GCTCATGTYTCHGGBAAAATWCG	[36]

## Data Availability

Not applicable.

## References

[1] Doebeli M., Knowlton N. (1998). The evolution of interspecific mutualisms. Proc. Natl. Acad. Sci. USA.

[2] Bomford B. (1966). The syngens of *Paramecium bursaria*: New mating types and intersyngenic mating reactions. J. Protozool..

[3] Witcherman R. (1985). The Biology of Paramecium.

[4] Greczek-Stachura M., Potekhin A., Przyboś E., Rautian M., Skoblo I., Tarcz S. (2012). Identification of *Paramecium bursaria* syngens through molecular markers—Comparative analysis of three loci in the nuclear and mitochondrial DNA. Protist.

[5] Hoshina R., Iwataki M., Imamura N. (2010). *Chlorella variabilis* and *Micractinium reisseri* sp. nov. (Chlorellaceae, Trebouxiophyceae): Redescription of the endosymbiotic green algae of *Paramecium bursaria* (Peniculia, Oligohymenophorea) in the 120th year. Phycol. Res..

[6] Gapanova I.N., Andronov E.E., Migunova A.V., Vorobyev K.P., Chizhevskaja E.P., Kvitko K.V. (2007). Genomic dactyloscopy of *Chlorella* sp., symbionts of *Paramecium bursaria*. Protistology.

[7] Pröschold T., Darienko T., Silva P.C., Reisser W., Krienitz L. (2011). The systematics of Zoochlorella revisited employing an integrative approach. Environ. Microbiol..

[8] Hoshina R., Imamura N. (2008). Multiple origins of the symbioses in *Paramecium bursaria*. Protist.

[9] Hoshina R., Imamura N., Fujishima M. (2009). Origins of algal symbionts of *Paramecium bursaria*. Endosymbionts in Paramecium. Microbiology Monographs.

[10] Hoshina R., Imamura N. (2009). Phyogenetically close group I introns with difefrent positions among *Paramecium bursaria* pfotobionts imply a primitive stage of intron diversification. Mol. Biol. Evol..

[11] Nakahara M., Handa S., Watanabe S., Deguchi H. (2004). *Choricystis minor* as a new symbiont of simultaneous two-species association with *Paramecium bursaria* and implications for its phylogeny. Symbiosis.

[12] Pröschold T., Pitsch G., Darienko T. (2020). *Micractinium tetrahymenae* (Trebouxiophyceae, Chlorophyta), a new endosymbiont isolated from ciliates. Diversity.

[13] Zagata P., Greczek-Stachura M., Tarcz S., Rautian M. (2016). The evolutionary relationships between endosymbiotic green algae of *Paramecium bursaria* syngens originating from different geographical locations. Folia Biol..

[14] Rosenberg J.N., Kobayashi N., Barnes A., Noel E.A., Btenbaugh M.J., Oyler G.A. (2014). Comparative analyses of three Chlorella species in response to light and sugar reveal distinctive lipid accumulation patterns in the microalga *C. sorokiniana*. PLoS ONE.

[15] Hong J.W., Jo S.W., Cho H.W., Nam S.W., Shin W., Park K.M., Lee K.I., Yoon H.S. (2015). Phylogeny, morphology, and physiology of *Micractinium* strains isolated from shallow ephemeral freshwater in Antarctica. Phycol. Res..

[16] Park K.C., Whitney C.G., Kozera C., O’Leary S.J., McGinn P.J. (2015). Seasonal isolation of microalgae from municipal wastewater for remediation and biofuel applications. J. App. Microbiol..

[17] Luo W., Pröschold T., Bock C., Krienitz L. (2010). Generic concept in Chlorella-related coccoid green algae (Chlorophyta, Trebouxiophyceae). Plant Biol..

[18] Kaplan-Levy R.N., Alster-Gloukhovski A., Benyamini Y., Zohary T. (2016). Lake Kinneret phytoplankton: Integrating classical and molecular taxonomy. Hydrobiologia.

[19] Darienko T., Gustavs L., Eggert A., Wolf W., Pröschold T. (2015). Evaluating the species boundaries of green microalgae (Coccomyxa, Trebouxiophyceae, Chlorophyta) using integrative taxonomy and DNA barcoding with further implications for the species identification in environmental samples. PLoS ONE.

[20] Caisova L., Marin B., Melkonian M. (2013). A consensus secondary structure of ITS2 in the Chlorophyta identified by phylogenetic reconstruction. Protist.

[21] Ustinova I., Krienitz L., Huss V.A.R. (2001). *Closteriopsis acicularis* (G.M. Smith) Belcher et Swale is a fusiform alga closely related to *Chlorella kessleri* Fott et Novakova (Chlorophyta, Trebouxiophyceae). Eur. J. Phycol..

[22] Nyberg D., Görtz H.D. (1988). The species concept and breeding systems. Paramecium.

[23] Jennings H.S. (1938). Sex reaction types and their interrelations in *Paramecium bursaria*: I. Proc. Natl. Acad. Sci. USA.

[24] Chen T.-T. (1956). Varieties and mating types in *Paramecium bursaria*. II. Variety and mating types found in China. J. Exp. Zool..

[25] Hoshina R., Hayashi S., Imamura N. (2006). Intraspecific genetic divergnce of *Paramecium bursaria* and re-construction of paramecian phylogentic tree. Acta Protozool..

[26] Zagata P., Greczek-Stachura M., Tarcz S., Rautian M. (2015). Molecular identification of *Paramecium bursaria* syngens and studies on geographic distribution using mitochondrial cytochrome C oxidase subunit I (*COI*). Folia Biol..

[27] Spanner C., Darienko T., Biehler T., Sonntag B., Pröschold T. (2020). Endosymbiotic green algae in *Paramecium bursaria*: A new isolation method and a simple diagnostic PCR approach for the identification. Diversity.

[28] Luo W., Pflugmacher S., Pröschold T., Walz N., Krienitz L. (2006). Genotype versus phenotype variability in *Chlorella* and *Micractinium* (Chlorophyta, Trebouxiophyceae). Protist.

[29] Weis D.S. (1978). Correlation of infectivity and concanavalin a agglutinability of algae exsymbiotic from *Paramecium bursaria*. J. Protozool..

[30] Reisser W., Vietze S., Widowski M. (1988). Taxonomic studies on endocytobiotic chlorophycean algae isolated from different American and European strains of *Paramecium bursaria*. Symbiosis.

[31] Meier R., Wiessner W. (1989). Infection of algae-free *Paramecium bursaria* with symbiotic *Chlorella* sp. isolated from green paramecia II: A time study. J. Cell Sci..

[32] Summerer M., Sonntag B., Sommaruga R. (2007). An experimental test of the symbiosis specificity between the ciliate *Paramecium bursaria* and strains of the unicellular green alga *Chlorella*. Environ. Microbiol..

[33] Summerer M., Sonntag B., Sommaruga R. (2008). Ciliate-symbiont specificity of freshwater endosymbiotic *Chlorella* (Trebouxiophyceae, Chlorophyta). J. Phycol..

[34] Fan W., Guo W., Van Etten J.L., Mower J.P. (2017). Multiple origins of endosymbionts in Chlorellaceae with no reductive effects on the plastid or mitochondrial genomes. Sci. Rep..

[35] Sonneborn T.M., Prescott E.D.M. (1970). Methods in *Paramecium* research. Methods in Cell Biology.

[36] Provan J., Murphy S., Maggs C.A. (2004). Universal plastid primers for Chlorophyta and Rodophyta. Eur. J. Phycol..

[37] White T.J., Bruns T., Lee S., Taylor J.W., Innis M.A., Gelfand D.H., Sninsky J.J., White T.M. (1990). Amplification and direct sequencing of fungal ribosomal RNA genes for phylogenetics. PCR Protocols: A Guide to Methods and Applications.

[38] Hoshina A.R., Kamako S.I., Imamura N. (2004). Phylogenetic position of endosymbiotic green algae in *Paramecium bursaria* Ehrenberg from Japan. Plant Biol..

[39] Librado P., Rozas J. (2009). DnaSP v5: A software for comprehensive analysis of DNA polymorphism data. Bioinformatics.

[40] Expertise in Software for Genetics aand Engineering. http://www.fluxus-engineering.com/.

[41] Bandelt H.J., Forster P., Röthl A. (1999). Median-Joining networks for inferring intraspecific phylogenies. Mol. Biol. Evol..

